# Micro-Mechanical Viscoelastic Properties of Crosslinked Hydrogels Using the Nano-Epsilon Dot Method

**DOI:** 10.3390/ma10080889

**Published:** 2017-08-02

**Authors:** Giorgio Mattei, Ludovica Cacopardo, Arti Ahluwalia

**Affiliations:** 1 Research Centre E. Piaggio, University of Pisa, Largo Lucio Lazzarino 1, 56122 Pisa, Italy; giorgio.mattei@centropiaggio.unipi.it (G.M.); ludovica.cacopardo@ing.unipi.it (L.C.); 2 Optics11 B.V., De Boelelaan 1081, 1081 HV Amsterdam, The Netherlands; 3 Biophotonics & Medical Imaging and LaserLaB, VU University Amsterdam, De Boelelaan 1105, 1081 HV Amsterdam, The Netherlands; 4 Department of Information Engineering, University of Pisa, Via Girolamo Caruso 16, 56122 Pisa, Italy

**Keywords:** nano-indentation, nano-epsilon dot method, strain rate, mechanical properties, viscoelastic models, soft materials, gelatin, glutaraldehyde

## Abstract

Engineering materials that recapitulate pathophysiological mechanical properties of native tissues in vitro is of interest for the development of biomimetic organ models. To date, the majority of studies have focused on designing hydrogels for cell cultures which mimic native tissue stiffness or quasi-static elastic moduli through a variety of crosslinking strategies, while their viscoelastic (time-dependent) behavior has been largely ignored. To provide a more complete description of the biomechanical environment felt by cells, we focused on characterizing the micro-mechanical viscoelastic properties of crosslinked hydrogels at typical cell length scales. In particular, gelatin hydrogels crosslinked with different glutaraldehyde (GTA) concentrations were analyzed via nano-indentation tests using the nano-epsilon dot method. The experimental data were fitted to a Maxwell Standard Linear Solid model, showing that increasing GTA concentration results in increased instantaneous and equilibrium elastic moduli and in a higher characteristic relaxation time. Therefore, not only do gelatin hydrogels become stiffer with increasing crosslinker concentration (as reported in the literature), but there is also a concomitant change in their viscoelastic behavior towards a more elastic one. As the degree of crosslinking alters both the elastic and viscous behavior of hydrogels, caution should be taken when attributing cell response merely to substrate stiffness, as the two effects cannot be decoupled.

## 1. Introduction

In their native environment, cells are surrounded by the extracellular matrix (ECM), a complex network of glycosaminoglycans, adhesion proteins, and structural fibers, serving not only as a physical scaffold, but also providing biochemical and biomechanical cues that are critical for the regulation of cell adhesion, proliferation, differentiation, morphology, and gene expression [1]. Among them, the ECM’s mechanical properties play a key role in directing cell fate and guiding pathophysiological cell behavior during tissue development, homeostasis, and disease [2,3,4,5]. Cells sense the mechanics of their surrounding environment (ECM) by gauging resistance to the traction forces they exert on it, and, in response, generate biochemical activity through a process known as mechano-transduction [6,7].

In the last few decades, several studies have focused on investigating the role of substrate elasticity (or stiffness) in cell mechano-transduction. A variety of biomaterials mimicking the native stiffness of different biological tissues have been proposed, particularly hydrogels (i.e., crosslinked three-dimensional (3D) networks of hydrophilic natural or synthetic polymers) [8]. Hydrogels have been widely used as cell culture substrates in mechano-transduction studies mostly because of their several advantages, such as high water content, biocompatibility, the availability of different crosslinking approaches, and high tunability, which allow recapitulation of the physicochemical and mechanical properties of native ECMs in vitro [9,10,11,12,13].

Studies on cell response to stiffness have significantly contributed to our understanding of cell mechano-transduction, designating substrate elasticity as a major determinant in the regulation of pathophysiological cell behavior and function [14,15]. For instance, stem cell commitment has been shown to depend on matrix elasticity [16], while tissue development, ageing, and disease progression are generally associated with tissue stiffening [17,18]. However, since native tissues [19,20] and hydrogels (e.g., gelatin [21,22], collagen [23], or fibrin [24]) typically exhibit viscoelastic behavior with stress-relaxation (i.e., a decrease in elastic modulus over time in response to a constant strain applied), focusing on stiffness only is generally an over-reductive way to describe their biomechanical properties. Moreover, culturing cells on primarily elastic substrates with constant (i.e., time-independent) elastic moduli is poorly representative of their native viscoelastic environment, where the resistance to the traction forces they exert is expected to relax over time due to flow and matrix remodeling [25].

There is thus a clear need to consider viscoelasticity when characterizing soft tissue biomechanics and developing biomaterials for cell culture and mechano-biology studies. To date, only a few studies have investigated the effect of substrate viscoelasticity on resultant cell behavior. Cameron and colleagues developed polyacrylamide gels with a shear loss modulus (G’’, reflecting the viscous component of the material viscoelastic behavior) varying over two orders of magnitude (from 1 to 130 Pa) and a nearly constant shear storage modulus (G” ~ 4.7 kPa, related to the elastic counterpart). In particular, increasing the substrate G’’ led to increased spreading and proliferation of human mesenchymal stem cells (hMSCs), but decreased the size and maturity of their focal adhesions, possibly because of decreased cytoskeletal tension resulting from the dissipation of energy owing to inherent substrate creep. Another recent study from Chauduri et al. investigated cell spreading on either almost elastic (i.e., covalently crosslinked) or viscoelastic (i.e., ionically crosslinked) alginate gels [25]. Despite the current consensus that cell spreading and proliferation are suppressed on soft substrates, they reported that cells cultured on soft viscoelastic substrates behave differently than those cultured on elastic substrates with the same initial elastic modulus, increasing spreading and proliferation to a similar extent as that observed on the stiffer elastic substrate. Taken together, these results suggest that stress-relaxation can compensate for the effect of decreased stiffness and has a substantial impact on cell behavior and function.

In light of the above considerations, it is natural to start wondering what is the best method to derive “physiologically relevant” viscoelastic properties (i.e., those describing the biomechanical environment felt by cells in their native milieu) to develop better mechano-mimetic cell culture substrates for tissue engineering, in vitro models, and mechano-transduction studies. Indeed, different mechanical properties (i.e., parameter values used to describe a given material’s mechanics) can be obtained when characterizing the same sample with different testing and analysis methods, likely leading to highly variable results that are difficult to interpret or not meaningfully comparable [18].

Among the available techniques, indentation testing with micron-sized probes is currently considered one of the most suitable methods for measuring a material’s mechanical properties at typical cell length-scales [26]. It requires minimal sample preparation, and allows mechanical mapping at multiple locations (e.g., to characterize local gradients and heterogeneities), thus it is particularly suited for most soft tissues and biomaterials [27,28,29]. We suggested that an ideal testing method for deriving physiologically relevant mechanical properties should (i) not require initial force- or strain-triggers (unlike dynamic mechanical analysis or step response tests, such as creep and stress-relaxation); and (ii) involve quick measurements, in order to avoid sample pre-stress and minimize status alterations during testing, respectively. Moreover, mechanical properties should be derived in the physiological region of small deformations (e.g., the 0.01 ÷ 0.1 strain range, depending on the tissue of interest), and measurements should be performed at physiologically relevant strain rates/frequencies (e.g., a 0.001 ÷ 0.1 s^−1^ strain rate) [18,20,21]. In this context, we recently proposed the nano-epsilon dot method (nano-$\dot{\varepsilon }M$) to characterize the physiologically relevant micro-mechanical viscoelastic properties of soft tissues and (bio)materials through nano-indentation tests at different constant strain rates (𝜀̇) [30]. Using data from the loading portion of the indentation curve and accurately identifying the initial point of contact, the nano-𝜀̇𝑀 allows for the derivation of “virgin” material viscoelastic properties (i.e., instantaneous and equilibrium elastic moduli as well as characteristic relaxation times) at typical cell length scales in the absence of pre-stress, unlike classical nano-indentation methods based on the analysis of the unloading curve (e.g., the Oliver–Pharr method [31,32]) or dynamic nano-indentation [33,34,35].

In this work, we used the nano-$\dot{\varepsilon }M$ to characterize the micro-mechanical viscoelastic properties of gelatin hydrogels. Gelatin is a low-cost, commercially available biomaterial derived from collagen, which is widely used as cell culture substrate mainly due to its inherent biocompatibility and bioactivity [36]. There are a variety of crosslinking strategies (e.g., chemical, enzymatic, and physical) available for improving its stability against enzymatic/hydrolytic degradation and tailoring its mechanical properties [37]. Glutaraldehyde (GTA) is one of the most widely used chemical crosslinking agents, particularly due to its highly efficient stabilization of collagenous materials through the reaction of free amino groups of lysine or the hydroxy-lysine amino acid residues of the polypeptide chains with its aldehyde groups [37,38]. Many studies have focused on characterizing the quasi-static elastic modulus (E) of GTA-crosslinked gelatin hydrogels, showing an increase in E with increasing GTA concentration [4,39]. However, as mentioned above, a single elastic modulus is an over-reductive way to describe the viscoelastic behavior of gelatin (and many other) hydrogels used in tissue engineering or cell culture applications. Therefore, the micro-mechanical viscoelastic properties of GTA-crosslinked gelatin hydrogels were characterized via nano-indentation tests, relating results to the crosslinker concentration.

## 2. Results

### 2.1. Apparent Elastic Moduli and Actual Sample Indentation Strain Rate

**Figure 1:**
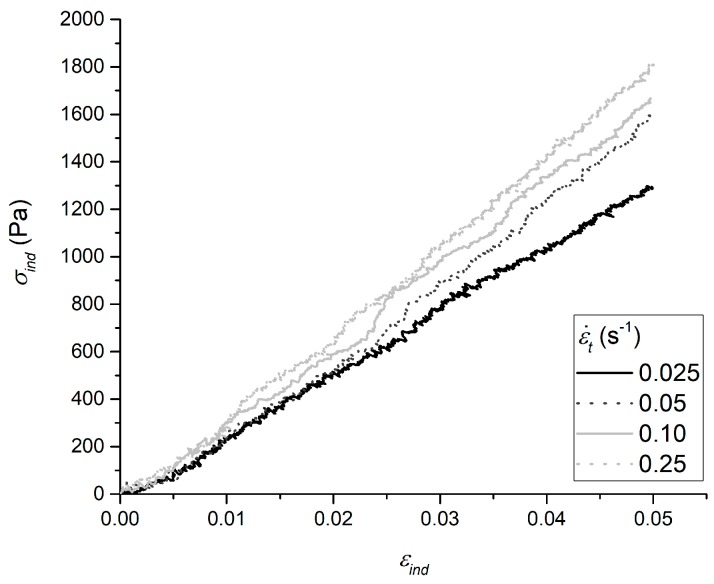
Examples of indentation stress-strain curves obtained testing 25 mM of GTA-crosslinked gelatin hydrogels. Sample viscoelasticity is reflected in the increase of apparent elastic modulus (i.e., stress versus strain slope) with increasing strain rate.

For all gelatin samples at different degrees of glutaraldehyde (GTA) crosslinking, experimental load-indentation (P-h) datasets collected at various constant theoretical strain rates (${\dot{\varepsilon }}_{t}$) were converted into indentation stress-strain ($\sigma_{ind}$-$\varepsilon_{ind}$) according to the nano-𝜀̇𝑀 definitions [30], as outlined in Section 4.2. The linear viscoelastic region (LVR) was found to extend up to $\varepsilon_{ind}$ = 0.05 for all samples and strain rates investigated (Figure 1).

The actual sample indentation strain rates (${\dot{\varepsilon }}_{ind}$) and strain rate-dependent “apparent” elastic moduli ($E_{app}$) obtained for the GTA-crosslinked gelatin hydrogels tested at different ${\dot{\varepsilon }}_{t}$ are summarized in Table 1.

The apparent elastic modulus ($E_{app}$) was found to increase with both increasing GTA concentration and strain rate, as expected due to the higher molar ratio between GTA aldehydes and gelatin free amino groups involved in the hydrogel chemical crosslink and because of the rate-dependent behaviour exhibited by viscoelastic materials, respectively. Notably, the actual sample indentation strain rate (${\dot{\varepsilon }}_{ind}$) was lower than the imposed theoretical indentation strain rate (${\dot{\varepsilon }}_{t}$). The difference between ${\dot{\varepsilon }}_{t}$ and ${\dot{\varepsilon }}_{ind}$ increases with $E_{app}$, as outlined in Section 4.2. Briefly, for a given cantilever with stiffness k (constant in all experiments), the higher the sample $E_{app}$, the higher the cantilever deflection rate ($\dot{d_{c}}$). This results in a lower sample indentation rate ($\dot{h}$) with respect to the piezo *z*-displacement rate set by the user ($\dot{d_{p}}$), and consequently in a lower ${\dot{\varepsilon }}_{ind}$ with respect to the ${\dot{\varepsilon }}_{t}$.

### 2.2. Maxwell Standard Linear Solid (SLS) Lumped Viscoelastic Constants

**Figure 2:**
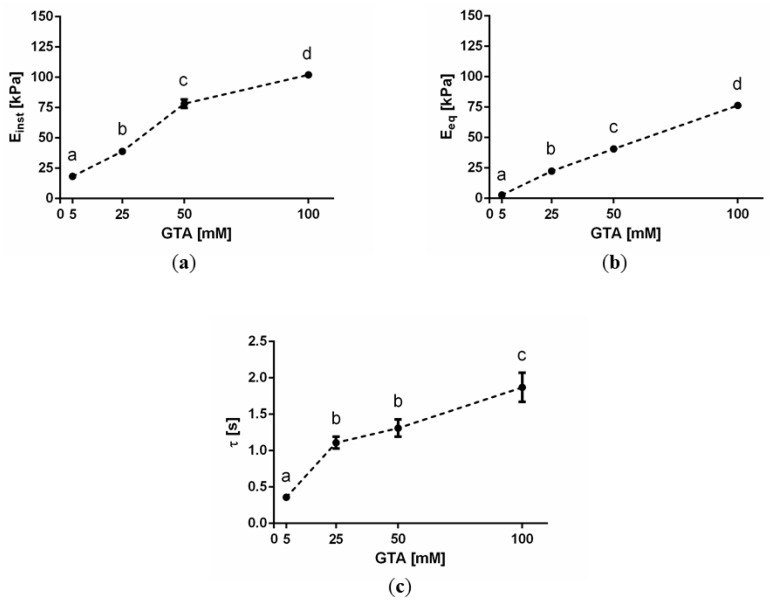
(**a**) Instantaneous ($E_{inst}$) and (**b**) equilibrium ($E_{eq}$) elastic moduli as well as (**c**) characteristic relaxation times (τ) as a function of glutaraldehyde (GTA) concentration obtained by globally fitting experimental nano-indentation stress-time data recorded at different constant strain rates to a Maxwell SLS lumped parameter model, as per the nano-$\dot{\varepsilon }M$. The error bars denote standard errors of estimation. Different letters indicate significant differences between samples (one-way ANOVA, *p* < 0.05), whereas the same letter means non-significant differences.

The Maxwell SLS viscoelastic parameters estimated through the nano-$\dot{\varepsilon }M$ global fitting procedure (Section 4.3) are reported as a function of GTA concentration in Figure 2, where $E_{inst}$ and $E_{eq}$ represent the instantaneous (i.e., $E_{0}+E_{1}$) and equilibrium ($E_{0}$) elastic moduli, respectively, while τ denotes the characteristic relaxation time calculated as $\eta_{1}/E_{1}$.

Both $E_{inst}$ and $E_{eq}$ significantly increased with GTA concentration (*p* < 0.0001). Moreover, a significant increase in τ. was also observed with increasing GTA concentration (*p* < 0.001), with the only exception between 25 and 50 mM GTA exhibiting statistically similar characteristic relaxation times (*p* = 0.66). Thus, the results obtained show that increasing the GTA concentration not only results in sample stiffening (reflected in the increased $E_{inst}$ and $E_{eq}$), but also changes the viscoelastic behaviour from a more viscous towards a more elastic one, as indicated by the longer τ. This viscoelasticity shift is also reflected in the lower strain rate dependency of the $E_{app}$ at higher GTA concentrations (Table 1).

## 3. Discussion

The results obtained in this study clearly show that characterizing sample stiffness only is generally an over-reductive way to describe the mechanical behaviour of GTA-crosslinked gelatin hydrogels. This consideration can be generalized to most soft biological tissues and hydrated biomaterials. We observed that gelatin hydrogels not only get stiffer with increasing GTA concentration, as demonstrated by increased instantaneous and equilibrium elastic moduli and as also expected from literature [4,39], but their viscoelastic behaviour concomitantly changes towards a more elastic one, as indicated by the longer relaxation time. The monotonic sample stiffening observed with increasing GTA concentration reflects an increase in the degree of crosslinking between gelatin free amino groups and GTA aldehydes. That the stiffness values do not reach a plateau suggests that the gelatin amines were not saturated [39].

Moreover, it is worth noting that the increase in elastic moduli with increasing GTA concentration obtained in this study using nano-indentation measurements (i.e., ~80 kPa in $E_{inst}$ and ~70 kPa in $E_{eq}$, from 5 to 100 mM GTA) is significantly higher than the increase in bulk elastic modulus we have observed performing unconfined compression tests on gelatin samples prepared in the same manner (i.e., ~20 kPa, from 5 to 100 mM GTA) [4]. This could be due to a number of factors, including:

(i) the scale-dependency of a sample’s mechanical properties, i.e., surface micro-mechanical properties could be different from bulk volumetric ones [40];
(ii) differences in testing and analysis methods, i.e., nano-indentation and unconfined compression techniques use different definitions of stress and strain, different models, etc., possibly affecting the mechanical properties obtained thereof [18]; and
(iii) sample volumetric heterogeneity, i.e., GTA-crosslinking might be not uniform within the gelatin hydrogel volume due to the passive diffusion-reaction mechanism, which is established when submerging physically crosslinked gelatin hydrogels in GTA solution. This may lead to a highly crosslinked hydrogel shell and less crosslinked core, resulting in a lower increase of bulk mechanical properties with increasing GTA [41].

The above considerations should warn researchers that the mechanical properties of a material are likely dependent on the testing length scale as well as the experimental and analysis methods used to derive them [18].

In conclusion, since hydrogel crosslinking generally results in a concomitant alteration of both elastic and viscous mechanical behavior, caution should be taken when attributing cell response to stiffness in these materials as the two effects interact and cannot be decoupled.

To the best of our knowledge, this is the first report on the characterization of gelatin viscoelastic properties as a function of GTA concentration using nano-indentation, showing that crosslinking not only increases stiffness, but also gives rise to an increase in the relaxation time and hence a shift from viscoelastic towards a more elastic behaviour. This shift is also likely to occur in pathophysiological processes such as organ development and fibrosis, suggesting a need to consider viscoelastic properties when characterizing biological tissue mechanics or engineering biomaterials for cell cultures.

## 4. Materials and Methods

### 4.1. Sample Preparation

A 5% w/v gelatin solution was prepared by dissolving type A gelatin powder (G2500, Sigma-Aldrich, Milan, Italy) in 1x phosphate buffered saline (PBS 1x, Sigma-Aldrich, Milan, Italy) at 50 °C under stirring for 2 h. Cylindrical gelatin samples with flat surfaces were obtained by casting the so-prepared solution in 8 mm height and 13 mm diameter molds, then leaving it to crosslink for 1 h at room temperature (RT). The physically gelled samples were removed from the molds and immersed for 48 h at 4 °C in glutaraldehyde (GTA; G5882, Sigma-Aldrich, Milan, Italy) crosslinking solutions prepared at different concentrations (i.e., 5, 25, 50, and 100 mM) in 40% v/v ethanol water solution, keeping a constant 5:1 volume ratio between the GTA solution and the gelatin samples to crosslink [4]. After chemical crosslinking, the samples were submerged in 0.5 M glycine solution (G7126, Sigma-Aldrich, Milan, Italy) for 2 h at RT to quench unreacted GTA, then copiously rinsed with deionized water. Finally, the samples were equilibrium swollen in PBS 1x at RT to be in a stable and reproducible state for testing [4,18,20] (equilibrium weight was reached within 1 h, data not shown), and mechanically characterized as described in the following sections.

### 4.2. Nano-Indentation Measurements

Equilibrium swollen samples were glued onto the bottom of a Petri dish and tested at different constant indentation strain rates in PBS 1x at RT according to the nano-$\dot{\varepsilon }M$ [30], using a displacement-controlled PIUMA Nanoindenter (Optics11 B.V., Amsterdam, The Netherlands). This instrument is based on a unique opto-mechanical ferrule-top cantilever force transducer operated by a *z*-axis piezoelectric motor, being very suited for nano-indentation measurements in liquids [42]. A probe having a 0.61 N/m cantilever stiffness (k) and a 70.5 µm radius (R) spherical tip was used in this study. The PIUMA Nanoindenter measures the indentation load as cantilever stiffness multiplied by its deflection resulting from pushing into the sample surface ($P=k\cdot d_{c}$), and calculates the actual indentation depth as the difference between the piezo *z*-axis displacement imparted to the probe and the resultant cantilever deflection ($h=d_{p}-d_{c}$). Thanks to the elastic nature of the cantilever, a constant piezo displacement rate results in a nearly constant “actual” sample indentation strain rate (${\dot{\varepsilon }}_{ind}$) within the region of small deformation, as outlined in Mattei et al. [30]. Since only the piezo displacement rate ($\dot{d_{p}}$) can be set by the user, the ${\dot{\varepsilon }}_{ind}$ is generally lower than the “theoretical” strain rate (${\dot{\varepsilon }}_{t}$) which would be obtained when using a cantilever with infinite stiffness (resulting in $d_{c}=0$, $h=d_{p}$, and $\dot{h}=\dot{d_{p}}$), and depends on the cantilever-to-sample stiffness ratio. In particular, the higher the cantilever-to-sample stiffness ratio, the lower the cantilever deflection rate ($\dot{d_{c}}$), the closer the actual sample strain rate (${\dot{\varepsilon }}_{ind}$) to the theoretical one (${\dot{\varepsilon }}_{t}$). Notably, with an infinite stiffness cantilever no load (P) can be measured. In this study, the total piezo *z*-displacement rate ($\dot{d_{p}}$) was set to obtain ${\dot{\varepsilon }}_{t}$ = 0.025, 0.05, 0.1, and 0.25 s^−1^ according to the following equation (Equation (1)) [30]:

(1) $$\dot{d_{p}}=\frac{3}{4}\cdot \left(1-\upsilon^{2}\right)\cdot R\cdot {\dot{\varepsilon }}_{t}$$

where υ is Poisson’s ratio, here assumed to be equal to 0.5 (i.e., incompressible material) for gelatin hydrogels [30,43,44]. Samples at different degrees of GTA-crosslinking were treated as mechanically isotropic and tested in triplicate (*n* = 3) performing *n* = 10 independent measurements per each strain rate investigated (i.e., a total of 40 tests per sample). Nano-indentation measurements were started out of sample contact and performed on different surface points (randomly selected) to avoid sample pre-stress and any eventual effect due to repeated testing cycles on the same spot, respectively.

### 4.3. Data Analyses and Viscoelastic Parameters Identification

Only data belonging to the loading portion of the load-indentation (P-h) curves measured at different ${\dot{\varepsilon }}_{t}$ were analyzed. The initial contact point was identified as the last one at which the load crosses the P-h abscissa towards monotonically increasing values [21,30]. Experimental P-h time data were offset to be zero in correspondence with this point. The load-indentation data were converted respectively into indentation stress ($\sigma_{ind}$) and strain ($\varepsilon_{ind}$) according to Equations (2) and (3) [30]:

(2) $$\sigma_{ind}=\frac{P}{R\cdot \sqrt{hR}}$$

(3) $$\varepsilon_{ind}=\frac{4}{3\cdot \left(1-\upsilon^{2}\right)}\cdot \frac{h}{R}$$

The linear viscoelastic region (LVR) was identified as the one in which $\sigma_{ind}$ increases linearly with $\varepsilon_{ind}$ ($R^{2}$ > 0.99), and strain-rate dependent “apparent” elastic moduli ($E_{app}$) were derived as the indentation stress-strain slope within the LVR. Then, the slope of the actual sample indentation strain rate (${\dot{\varepsilon }}_{ind}$) was calculated as the slope of experimental strain ($\varepsilon_{ind}$) versus time (t) within the LVR, and used for the nano-$\dot{\varepsilon }M$ lumped viscoelastic parameters’ identification [30]. In particular, the Maxwell Standard Linear Solid model (SLS) was chosen to represent the viscoelastic behavior of gelatin in this work [21,30,45,46]. The SLS model is the simplest form of the Generalized Maxwell lumped parameter model. It consists of a pure spring ($E_{0}$) assembled in parallel to a Maxwell arm (i.e., a spring $E_{1}$ in series with a dashpot $\eta_{1}$, defining a characteristic relaxation time $\tau_{1}=\eta_{1}/E_{1}$) [47], and exhibits the following stress-time response to a constant indentation strain rate input ${\dot{\varepsilon }}_{ind}$ (Equation (4)) [21,30]:

(4) $$\sigma_{ind}\left(t\right)={\dot{\varepsilon }}_{ind}\cdot \left(E_{0}t+\eta_{1}\left(1-e^{-\frac{E_{1}}{\eta_{1}}t}\right)\right)$$

For each gelatin sample at a different GTA-crosslinking grade, experimental stress-time series within the LVR obtained at different indentation strain rates were globally fitted to Equation (4) for deriving the Maxwell SLS viscoelastic constants (i.e., $E_{0}$, $E_{1}$, and $\eta_{1}$). The global fitting procedure was implemented in OriginPro (OriginLab Corp., Northampton, MA, USA), performing chi-square minimization in a combined parameter space. In particular, for each set of stress-time data considered in the global fitting, the ${\dot{\varepsilon }}_{ind}$ value of the fitting equation (Equation (4)) was set to be equal to the actual one calculated from the experiments (i.e., $\varepsilon_{ind}$ vs t slope), while the SLS viscoelastic constants to estimate were shared between datasets.

An annealing scheme based on multiplying and dividing each initial parameter guess by 10 while keeping the instantaneous modulus (i.e., $E_{inst}=E_{0}+E_{1}$) at a constant value was adopted to obtain reliable and absolute SLS viscoelastic constant estimations, avoiding most of the local minima during the fitting procedure. Viscoelastic constants to estimate were constrained to be ≥0 to prevent the fitting procedure returning negative values.

### 4.4. Statistical Analyses

Results are reported as mean ± standard error (unless otherwise noted). Statistical differences between viscoelastic parameters of gelatin hydrogels at different GTA-crosslinking grades were tested using one-way ANOVA followed by Tukey’s Multiple Comparison Test. Statistical analyses were performed in GraphPad Prism (GraphPad Software, San Diego, CA, USA), setting significance at *p* < 0.05.

## Figures and Tables

**Table 1:** Actual indentation strain rates (${\dot{\varepsilon }}_{ind}$) and apparent elastic moduli ($E_{app}$) obtained for GTA-crosslinked samples tested at different theoretical strain rates (${\dot{\varepsilon }}_{t}$). Values are reported as mean ± standard error.

GTA (mM)	${\dot{\varepsilon }}_{t}$ (s^−1^)	$E_{app}$ (kPa)	${\dot{\varepsilon }}_{ind}$ (s^−1^)
5	0.025	5.3 ± 0.3	0.021 ± 0.001
	0.05	9.3 ± 0.8	0.047 ± 0.001
	0.10	12.4 ± 0.6	0.070 ± 0.001
	0.25	17.3 ± 1.1	0.150 ± 0.001
25	0.025	27.5 ± 0.6	0.012 ± 0.001
	0.05	30.9 ± 2.1	0.024 ± 0.001
	0.10	35.2 ± 0.8	0.044 ± 0.001
	0.25	37.3 ± 0.9	0.124 ± 0.001
50	0.025	53.9 ± 0.8	0.008 ± 0.001
	0.05	57.8 ± 0.6	0.016 ± 0.001
	0.10	62.9 ± 0.2	0.031 ± 0.001
	0.25	65.3 ± 1.9	0.098 ± 0.001
100	0.025	76.7 ± 2.9	0.006 ± 0.001
	0.05	79.7 ± 1.3	0.013 ± 0.001
	0.10	83.0 ± 1.3	0.025 ± 0.001
	0.25	84.8 ± 1.1	0.067 ± 0.001
